# Supplementary material for: Simultaneous quantification of mRNA and protein in single cells reveals post-transcriptional effects of genetic variation
Source: eLife. 2020 Nov 16;9:e60645. doi: 10.7554/eLife.60645 (PMC7707838; doi:10.7554/eLife.60645)
Supplement: Transparent reporting form [file elife-60645-transrepform.docx]

***eLife’s* transparent reporting form**

We encourage authors to provide detailed information *within their submission* to facilitate the interpretation and replication of experiments. Authors can upload supporting documentation to indicate the use of appropriate reporting guidelines for health-related research (see [EQUATOR Network](http://www.equator-network.org/%20)), life science research (see the [BioSharing Information Resource](https://biosharing.org/)), or the [ARRIVE guidelines](http://www.plosbiology.org/article/info:doi/10.1371/journal.pbio.1000412) for reporting work involving animal research. Where applicable, authors should refer to any relevant reporting standards documents in this form.

If you have any questions, please consult our Journal Policies and/or contact us: [editorial@elifesciences.org](mailto:editorial@elifesciences.org).

**Sample-size estimation**

- You should state whether an appropriate sample size was computed when the study was being designed
- You should state the statistical method of sample size computation and any required assumptions
- If no explicit power analysis was used, you should describe how you decided what sample (replicate) size (number) to use

Please outline where this information can be found within the submission (e.g., sections or figure legends), or explain why this information doesn’t apply to your submission:

We did not conduct power analyses specifically for this study. However, our bulk segregant QTL mapping approach is explicitly designed to have high power, as demonstrated in multiple prior publications that are cited in the manuscript.

For allele swaps, reporter characterization, and differential expression analyses, we chose sample numbers by considering practical constraints of time and resources, practice in the field, and our own experience.

**Replicates**

- You should report how often each experiment was performed
- You should include a definition of biological versus technical replication
- The data obtained should be provided and sufficient information should be provided to indicate the number of independent biological and/or technical replicates
- If you encountered any outliers, you should describe how these were handled
- Criteria for exclusion/inclusion of data should be clearly stated
- High-throughput sequence data should be uploaded before submission, with a private link for reviewers provided (these are available from both GEO and ArrayExpress)

Please outline where this information can be found within the submission (e.g., sections or figure legends), or explain why this information doesn’t apply to your submission:

This information is available throughout the manuscript in figure legends, Results and Materials & Methods. Biological and technical replicates are clearly defined in the Methods. Replicate numbers are clearly visible in Figures, or stated in the legends of figures 1, 2, 4, and 6.

Raw reads and transcriptome data are currently being uploaded to SRA and GEO, respectively, via BioProject PRJNA644804: <https://www.ncbi.nlm.nih.gov/bioproject/644804>

**Statistical reporting**

- Statistical analysis methods should be described and justified
- Raw data should be presented in figures whenever informative to do so (typically when N per group is less than 10)
- For each experiment, you should identify the statistical tests used, exact values of N, definitions of center, methods of multiple test correction, and dispersion and precision measures (e.g., mean, median, SD, SEM, confidence intervals; and, for the major substantive results, a measure of effect size (e.g., Pearson's r, Cohen's d)
- Report exact p-values wherever possible alongside the summary statistics and 95% confidence intervals. These should be reported for all key questions and not only when the p-value is less than 0.05.

Please outline where this information can be found within the submission (e.g., sections or figure legends), or explain why this information doesn’t apply to your submission:

Raw data for individual replicates is plotted throughout the paper, in particular in Figures 1, 2, 4, and 6. Full measurements for all 5 (per group) mass spectrometry replicates for proteomics on the YAK1 variant are available as Source Data for Figure 7. Expression values for the RNASeq measures are available on GEO (see above). Effect sizes and exact p-values for correlations and various statistical tests are presented throughout the Results. Full QTL mapping results with replication information, information on individual LOD scores and effect sizes is provided as Source Data for Figure 4. Detailed statistical results for mass spectrometry and RNA sequencing are available as Source Data for Figure 7. Other statistical tests are indicated in Figures 6 and 7, in some cases in the legends.

(For large datasets, or papers with a very large number of statistical tests, you may upload a single table file with tests, Ns, etc., with reference to sections in the manuscript.)

**Group allocation**

- Indicate how samples were allocated into experimental groups (in the case of clinical studies, please specify allocation to treatment method); if randomization was used, please also state if restricted randomization was applied
- Indicate if masking was used during group allocation, data collection and/or data analysis

Please outline where this information can be found within the submission (e.g., sections or figure legends), or explain why this information doesn’t apply to your submission:

For all pairwise comparisons of individual alleles or constructs, we pseudo-randomized the samples such that each allele was equally represented in any given measurement batch. This information is available in Materials and Methods. Our QTL mapping procedure is randomized by design, since we do not know the genotype of individual cells when we sort them.

**Additional data files (“source data”)**

- We encourage you to upload relevant additional data files, such as numerical data that are represented as a graph in a figure, or as a summary table
- Where provided, these should be in the most useful format, and they can be uploaded as “Source data” files linked to a main figure or table
- Include model definition files including the full list of parameters used
- Include code used for data analysis (e.g., R, MatLab)
- Avoid stating that data files are “available upon request”

Please indicate the figures or tables for which source data files have been provided:

All analysis code is available at: <https://github.com/BrionChristian/Simultaneous_RNA_protein_QTLs>

Source data are provided for Figures 4, 5, and 7. No data are “available upon request”; all data is freely available with the paper or on github/GEO/SRA.
